# Acute ST-segment Elevation Myocardial Infarction as the First Manifestation of Essential Thrombocytosis

**DOI:** 10.7759/cureus.4032

**Published:** 2019-02-07

**Authors:** Purva Sharma, Sameer Gupta, Pankit Patel, Yuanming Zhang, Shachar Peles

**Affiliations:** 1 Internal Medicine, University of Miami Miller School of Medicine, Atlantis, USA; 2 Pathology, JFK Medical Center, Boca Raton, USA; 3 Oncology, Florida Cancer Specialists, Atlantis, USA

**Keywords:** essential thrombocytosis, thrombosis, coronary artery disease, myocardial infarction, myeloproliferative disorders

## Abstract

A 36-year-old female with no significant past medical history presented with sudden onset of crushing substernal chest pain. When the emergency medical services (EMS) arrived, she had a cardiac arrest requiring defibrillation two times in the field prior to arriving at the hospital. In the emergency department (ED), the electrocardiogram (ECG) was significant for ST-elevation that suggested acute anterolateral infarct. Her laboratory evaluation also showed a platelet count of 1095 x 10^3^/ul. Also, her troponin levels were at 0.16 ng/ml at the time of arrival and peaked at 42.8 ng/ml. She immediately underwent a cardiac catheterization which showed 100% occlusion of her left anterior descending (LAD) artery by a thrombus, which was then treated with a thrombectomy and a single drug-eluting stent was placed. Upon further work-up of her thrombocytosis, the patient had a bone marrow biopsy showing megakaryocytic hyperplasia which no evidence of fibrosis. She was tested for Janus kinase 2 (JAK2) mutation which was positive. The patient was diagnosed with essential thrombocytosis (ET) and was started on cytoreduction therapy with hydroxyurea. Her platelet counts responded appropriately and dropped to less than 500 x 10^3^ at the time of discharge.

## Introduction

Essential thrombocytosis (ET) is a chronic myeloproliferative disorder associated with an increased tendency to thrombosis [1]. The high platelet count predisposes patients to complications such as coronary artery thrombosis, even in the absence of significant risk factors. We present the case of a 36-year-old patient with no conventional cardiovascular risk factors, who presented with an ST-segment elevation myocardial infarction (STEMI) and was finally diagnosed as a case of ET.

## Case presentation

**Figure 1:**
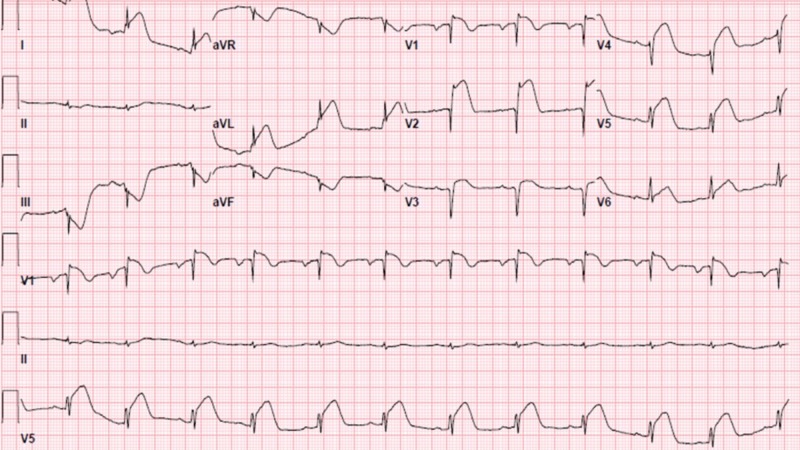
12-lead electrocardiogram (ECG) showing ST elevation in leads V1, V2, and V3

A 36-year-old, previously healthy Caucasian woman, presented with complaints of sudden onset of chest pain. The patient described the pain as crushing, pressure-type, located in the sub-sternal region and radiating to the left arm. The pain started while she was showering in the morning, and after the shower, she felt light headed and called the emergency medical services (EMS). The patient has a 20 pack-year history of smoking. Upon arrival of the EMS, the patient went into cardiac arrest with ventricular fibrillation. She required defibrillation two times in the field with successful return of spontaneous circulation; she was then brought to the emergency department (ED). In the ED, the patient's electrocardiogram (ECG) showed ST-segment elevations in the anterior leads with ST-segment depression inferiorly with reciprocal changes (Figure 1).

Her laboratory evaluation was also significant for hemoglobin at 16.5 g/dL, hematocrit at 50.3%, and a platelet count of 1095 x 10^3^/ul. She was hypokalemic with a potassium of 3.0 mmol/L, HCO3 was low at 16 mmol/L with an anion gap of 18. Her troponin level was elevated at 0.16 ng/ml initially and later peaked to 42.8 ng/ml.

**Figure 2:**
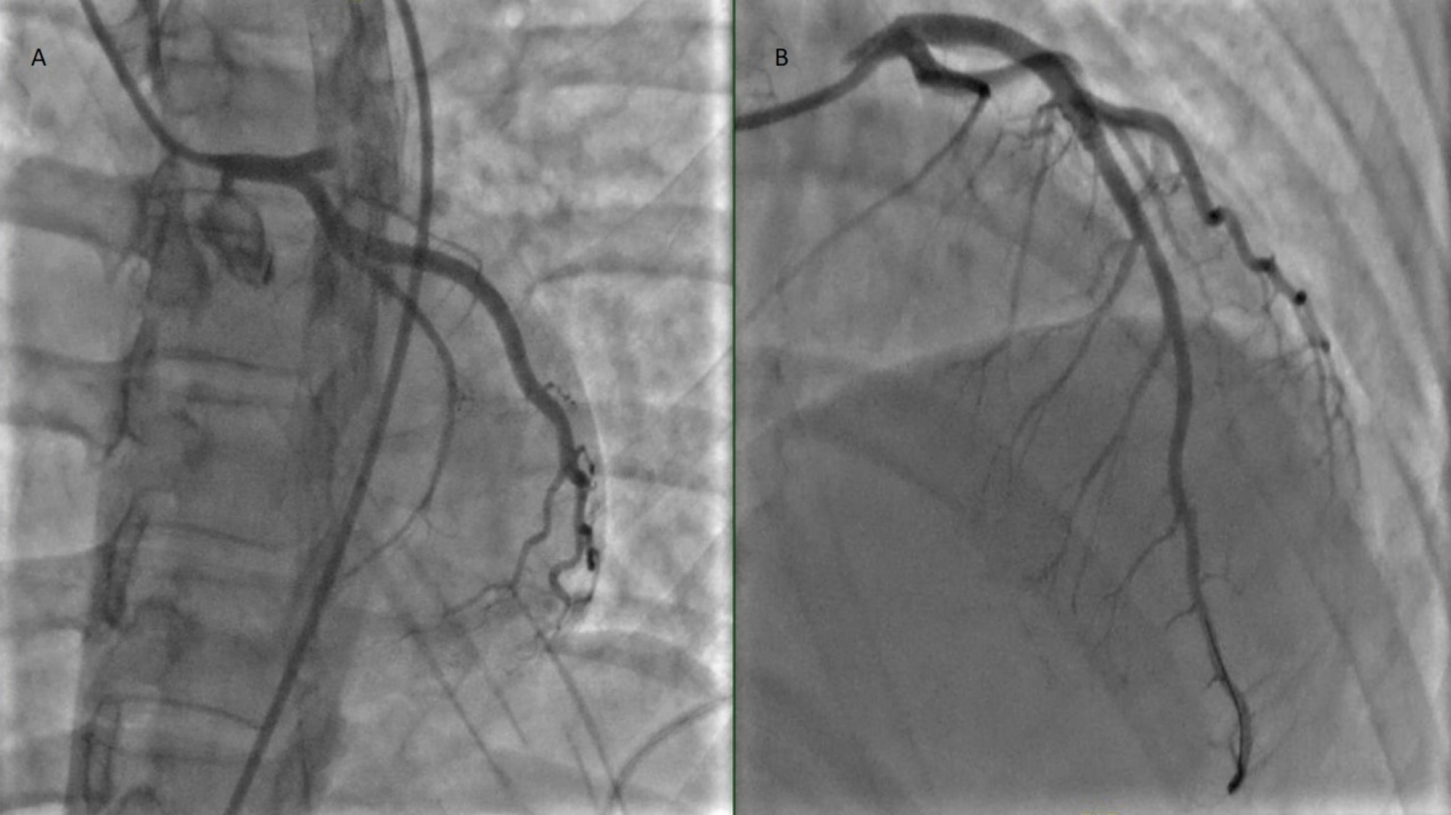
Angiogram showing A) 100% occlusion of the left anterior descending (LAD) artery, B) drug-eluting stent placement in the LAD artery post thrombectomy

The patient was immediately taken to the cardiac catheterization lab and coronary angiography was performed which revealed 100% occlusion in the proximal left anterior descending (LAD) artery with thrombus. The thrombus was treated with intra-coronary eptifibatide; AngioJet (Boston Scientific Corp., Natick, MA) aspiration thrombectomy was performed with placement of a single drug-eluting stent in the proximal LAD (Figure 2).

**Figure 3:**
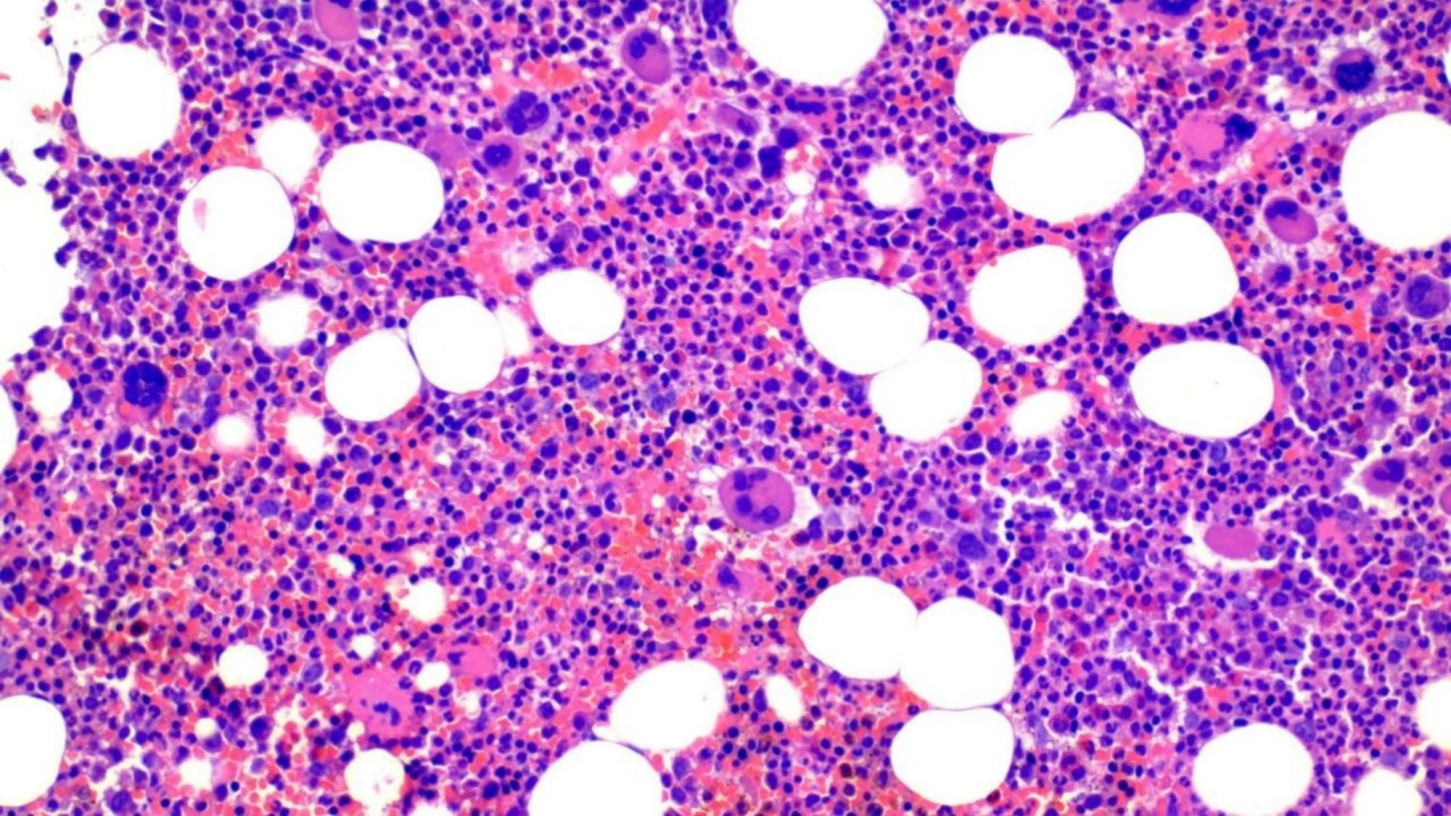
Bone marrow biopsy showing megakaryocytic hyperplasia with no evidence of fibrosis

The patient did well post-procedure without any major complications. Work up for patient's thrombocytosis was initiated. Microscopic evaluation of peripheral smear was notable for thrombocytosis and erythrocytosis in the blood. Platelet function collagen/epi and adenosine diphosphate (ADP) were high. At this stage, as a myeloproliferative neoplasm was suspected, the patient was started on cytoreduction therapy with hydroxyurea. She was also tested for BCR/ABL1 gene re-arrangement, which was found to be normal. The patient's Janus kinase 2 (JAK2) V617F mutation testing was positive which increased suspicion for ET, with a small possibility of primary myelofibrosis. She also had a bone marrow biopsy which showed normocellular bone marrow with myeloid and megakaryocytic hyperplasia and no increase in reticulin fibrosis (Figure 3).

The morphologic findings in conjunction with the positive JAK2 V617F mutation analysis of the peripheral blood specimen confirmed the diagnosis of a myeloproliferative neoplasm. The differential diagnosis includes ET versus early stage/prefibrotic stage of primary myelofibrosis (PMF). The overall findings favoured ET.

The patient continued to be on hydroxyurea with a gradual fall in platelet count. She did not require plateletpheresis or a second agent to lower her cell count.

## Discussion

ET is one of the chronic myeloproliferative neoplasms characterized by excessive, clonal platelet production leading to complications of thrombosis and hemorrhage. Thrombotic complications in ET can be arterial or venous and include strokes, transient ischemic attacks, coronary artery ischemia, pulmonary embolisms or deep vein thrombosis. Patients are also at increased risk of bleeding, especially when platelet counts are more than 1.5 million due to likely platelet dysfunction.

The incidence of acute coronary events in patients with ET is reported to be around 9.4% and increases with age [2]. Median age at diagnosis of ET is in the sixth decade of life, with less than 20% of patients being diagnosed below 40 years [3]. Studies have shown that there is a female preponderance in ET as well [2].

Acute coronary syndrome constitutes a major cause of morbidity and mortality in patients with untreated ET. In a study by Gao et al., it was reported that LAD was the most commonly occluded artery in patients with myocardial infarction (MI) and ET [4-5].

The mutation of JAK2 V617F is present in 50% to 60% of ET cases [5]. This mutation increases the risk of thrombosis by approximately two-fold in patients with ET. It has been reported that hypertension and smoking are risk factors for the development of thrombosis in these patients [5]. In a study evaluating the factors associated with higher risk of thrombosis in 126 young patients (between the ages of five and 40 years) with ET, it was reported that smoking was the only predictive factor, with the actuarial probability of thrombosis being 72% at 10 years in smokers [6].

Treatment with hydroxyurea has been shown to reduce the risk of thrombosis in high-risk (age >60 or history of thrombosis) ET patients with a goal platelet count of 400,000/microL [4]. Aggressive treatment with antiplatelet agents in the presence of ET is recommended to minimize the risk of thromboembolic events.

## Conclusions

ET is one of the myeloproliferative neoplasms that is, more often than not, diagnosed incidentally when thrombocytosis is noted on a complete blood count. If left undiagnosed, it can have life-threatening consequences of thrombosis such as stroke, coronary artery ischemia or severe hemorrhage. Patients can sometimes present with one of the life-threatening consequences of ET as the first manifestation of the disease. It is therefore important to rule out an underlying myeloproliferative neoplasm as a cause of arterial/venous thrombotic event, especially in younger patients, at the time of diagnosis.
